# Ärztliche Aufklärungspflichten nach Bekanntgabe eines Warnhinweises über unerwünschte Arzneimittelwirkungen

**DOI:** 10.1007/s00063-020-00656-5

**Published:** 2020-02-07

**Authors:** G. Duttge, T. Meyer

**Affiliations:** 1 Zentrum für Medizinrecht, Georg-August-Universität Göttingen, Göttingen, Deutschland; 2 Klinik für Psychosomatische Medizin und Psychotherapie, Georg-August-Universität Göttingen, Waldweg 33, 37073 Göttingen, Deutschland

**Keywords:** Hormonelle Kontrazeption, Suizidalität, Depression, Selbstbestimmungsaufklärung, § 630e Bürgerliches Gesetzbuch, Fach- und Gebrauchsinformationen für Arzneimittel, Hormonal contraception, Suicidality, Depression, Acquiring informed consent, § 630e German Civil Code, Summary of product characteristics/package leaflets

## Abstract

**Hintergrund:**

Ausgehend von einem aktuellen Warnhinweis der Arzneimittelbehörden über eine erhöhte Suizidalität bei Einnahme hormoneller Kontrazeptiva werden in diesem Artikel die juristischen Konsequenzen der Umsetzung von neuen, aus klinischen Studien gewonnenen Erkenntnissen in geänderte Inhalte von ärztlichen Aufklärungsgesprächen diskutiert.

**Methode und Ergebnisse:**

Die nach § 630e Bürgerliches Gesetzbuch (BGB) gebotene ärztliche Aufklärung verlangt grundsätzlich auch die Erwähnung seltener Arzneimittelwirkungen durch den behandelnden Arzt, sollten die verordneten Medikamente im Einzelfall schwerwiegende medizinische Folgen nach sich ziehen. Die Vorschrift sieht eine Aufklärungsverpflichtung des Behandelnden nicht nur bei kurativen, sondern auch bei ausschließlich präventiven Behandlungsmaßnahmen vor. In dieser Arbeit weisen wir auf die wissenschaftliche Evidenzlage von klinischen Studienergebnissen als der entscheidenden Vorbedingung für die Implementierung von geänderten Praktiken bei der ordnungsgemäßen Durchführung einer haftungsausschließenden Selbstbestimmungsaufklärung nach § 630e BGB hin. Zugleich werden die Auswirkungen einer Ergänzung der Gebrauchs- bzw. Fachinformationen für Arzneimittel auf den Umfang der Aufklärungspflichten des im individuellen Fall verordnenden Arztes thematisiert. Im Besonderen wird das Verhältnis von ärztlichen Aufklärungspflichten im individuellen Aufklärungsfall zu den generalisierten Informationsgegebenheiten nach § 11 bzw. § 11a Arzneimittelgesetz hervorgehoben.

**Diskussion:**

Aktuelle Warnhinweise der Arzneimittelbehörden in Form von Rote-Hand-Briefen haben nicht zwingend juristische Konsequenzen für die Inhalte von ärztlichen Aufklärungsgesprächen.

## Abstract (en)

**Background:**

Based on a recent warning from the drug authorities about increased suicidality among users of hormonal contraceptives, this article discusses the legal consequences of translating novel findings from clinical trials into altered contents of gaining informed consent during the medical consultation.

**Methods and results:**

Comprehensive information in accordance with § 630e German Civil Code (BGB) requires that rare drug reactions be mentioned by the prescribing physician, when they are associated with serious sequelae. This act regulates the treating physician’s obligations to inform about complications for both preventive and curative treatment options. In this paper, we refer to the scientific evidence level of data from clinical trials as the key feature for implementing altered medical information in the proper conduct of acquiring the consent of the patient in line with § 630e BGB. The article discusses how additions and amendments to the package leaflet and the expert information will impact on the obligations for the treating party to provide information for the patient in order to obtain informed consent. In particular, we focus on the relationship between the obligations for the physician prescribing oral contraceptives on an individual case and the generalized information conditions according to § 11 and § 11a German Medicinal Products Act.

**Discussion:**

Current warnings of the drug authorities in the form of red-hand letters do not necessarily have legal consequences for gaining informed consent during the medical consultation.

## Aktueller Warnhinweis für ein erhöhtes Suizidrisiko bei Anwendung hormoneller Kontrazeptiva

In Absprache mit dem Bundesinstitut für Arzneimittel- und Medizinprodukte (BfArM) warnten in einem im Januar 2019 veröffentlichten Rote-Hand-Brief 35 deutsche Zulassungsinhaber und Vertreiber von hormonellen Kontrazeptiva vor einem erhöhten Suizidrisiko bei bestimmungsgemäßem Gebrauch von oralen Verhütungsmitteln. Die in dieser Arzneimittelinformation geäußerten Sicherheitsbedenken ergaben sich aus Empfehlungen des Ausschusses für Risikobewertung im Bereich der Pharmakovigilanz (PRAC) bei der Europäischen Arzneimittelagentur (EMA), die zuvor ein Signalverfahren zu hormonellen Kontrazeptiva aufgrund des Verdachts auf ein möglicherweise erhöhtes Risiko von Suizidversuchen und Suiziden abgeschlossen hatte und sich dabei maßgeblich auf die Ergebnisse einer prospektiven dänischen Kohortenstudie berief. Der Rote-Hand-Brief behauptete einen Zusammenhang zwischen dem Auftreten depressiver Verstimmungen bzw. depressiver Syndrome und der Anwendung von hormonellen Kontrazeptiva, der bereits zum etablierten medizinischen Wissen gehöre, ohne dass dieses allerdings durch Literaturangaben belegt wurde. In dem Warnhinweis wird zudem eine Verbindung zwischen einer erhöhten Suizidrate und einer durch die Einnahme von hormonellen Kontrazeptiva induzierten erhöhten Depressivität unterstellt. Obgleich die Arzneimittelinformation zwar einschränkend einen Kausalzusammenhang zwischen der Einnahme von hormonellen Kontrazeptiva und einem Anstieg der Suizidrate nicht für eindeutig bewiesen hält, soll aber der ähnlich gelagerte Zusammenhang mit einer erhöhten Depressivität eine solche Vermutung nahelegen.

Ausdrücklich verweist das Schreiben deshalb auf die Empfehlung der EMA zur entsprechenden Aufnahme einer Warnung in die Fach- und Gebrauchsinformation und enthält zusätzlich einen Vermerk zur stärkeren Sensibilisierung von Angehörigen der Heilberufe, die ihre Patientinnen bei der Verschreibung hormoneller Verhütungsmittel ergänzend über Stimmungsschwankungen aufzuklären haben. Zu den Folgen für das Aufklärungsgespräch im Einzelnen macht der Rote-Hand-Brief allerdings keine Vorgaben.

In dem vorliegenden Artikel soll deshalb nach kurzer Schilderung der gegenwärtigen Studienlage unter Einbeziehung auch eigener Ergebnisse einer Post-hoc-Analyse zur Lebensqualität jugendlicher Anwenderinnen von oralen Kontrazeptiva die medizinrechtlich relevante Frage diskutiert werden, welche juristischen Konsequenzen sich aus der Publikation eines solchen Warnhinweises ergeben. Dabei wird anhand des aktuellen Beispiels einer ärztlichen Verschreibung von hormonenthaltenden Verhütungsmitteln ein kurzer Überblick über die normativen Voraussetzungen und die aktuelle Rechtsprechung zur spezifischen arzneimittelbezogenen Aufklärungspflicht der behandelnden Ärzte gegeben.

## Studien zum Zusammenhang von hormonaler Kontrazeption, Suizidalität und Depressivität

Nicht nur für psychiatrisch tätige Fachärzte stellt die Bewältigung von akuten suizidalen Krisen eine große Herausforderung dar, sondern immer häufiger werden auch Rettungsmediziner und Notfallärzte auf internistischen Intensivstationen mit der Erstversorgung von lebensbedrohlichen Komplikationen einer Suizidhandlung konfrontiert, sofern diese akute Episode von den Patienten überlebt wird. Auf die grundlegende Bedeutung der medizinisch-psychologischen Behandlung suizidaler Patienten in der Notfallmedizin wiesen Flüchter und Kollegen in einer wichtigen Publikation, erschienen in *Medizinische Klinik – Intensivmedizin und Notfallmedizin*, hin und betonten dabei insbesondere die gesellschaftlichen, medizinischen und rechtlichen Aspekte im Umgang mit den schwer erkrankten Überlebenden von Suizidversuchen [6]. Wenn die Einnahme von oralen Kontrazeptiva tatsächlich mit einer erhöhten Suizidalität verbunden sein sollte, ergäben sich vielfältige Implikationen für die internistische Intensiv- und Notfallmedizin, die vom Erkennen eines solchen Zusammenhangs bis hin zur unmittelbaren Behandlung reichen würden. Da orale Kontrazeptiva als eine zuverlässige Verhütungsmethode sehr häufig verschrieben werden, könnte dieses unter epidemiologischen Gesichtspunkten für internistisch tätige Notfall- und Rettungsmediziner von erheblicher praktischer Relevanz sein.

Neben der Indikation zur Verhinderung ungewollter Schwangerschaften werden hormonelle Kontrazeptiva bei Frauen im gebärfähigen Alter auch zur Behandlung von Menstruationsschmerzen, Symptomen im Zusammenhang mit dem prämenstruellen Syndrom und Hypermenorrhöen sowie zur Linderung von Akne vulgaris eingesetzt. Seit ihrer Einführung auf dem deutschen Markt im Jahr 1961 verfügen wir über langjährige Erfahrungen im Umgang mit oralen Hormonpräparaten, die zu der mit am häufigsten verordneten Arzneimittelklasse zählen. Trotz der langjährigen Erfahrungen mit östrogen- und progesteronhaltigen Kontrazeptiva sind klinische Studien zu ihrem Einfluss auf psychische Gesundheit und gesundheitsbedingte Lebensqualität selten und die wenigen Studien hierzu überdies in ihren Aussagen widersprüchlich [3, 14, 16, 18, 21]. Insbesondere bei Jugendlichen und jungen Frauen wurde der Einfluss von exogenen Geschlechtshormonen auf die Auslösung depressiver Symptome kontrovers diskutiert, und es ist nicht geklärt, ob eine erhöhte Inzidenz von depressiven Syndromen bzw. das Entstehen von Stimmungsschwankungen als eine klinisch relevante Nebenwirkung von hormonbasierter Kontrazeption bei der Verschreibung als Verhütungsmittel tatsächlich auftritt [2, 8, 22].

Eine große US-amerikanische Studie, an der 6654 sexuell aktive Frauen im Alter von 23–34 Jahren teilnahmen, berichtete sogar im Gegenteil, dass hormonelle Verhütung das Ausmaß depressiver Symptome bei jungen Frauen senken kann und somit der Verwendung von Hormonpräparaten eine gewisse protektive Funktion gegenüber dem Auftreten von depressiven Verstimmungen zukomme [10]. Ähnliche Ergebnisse wurden auch von einer kleinen norwegischen Querschnittsstudie mit erwachsenen Frauen veröffentlicht, die eine verringerte Wahrscheinlichkeit für das Auftreten von Stimmungsstörungen bei den Konsumentinnen von kombinierten Verhütungspräparaten nahelegte, wohingegen die Anwendung von ausschließlich progesteronhaltigen Kontrazeptiva eine schädliche Wirkung zeigte [19]. Eine kleine Studie mit jungen Frauen im Alter von 14–20 Jahren ergab keine signifikanten Veränderungen bei der selbsteingeschätzten Lebensqualität oder der Entstehung von depressiven Symptomen, doch wurden sowohl die Anzahl der Tage mit Menstruationsblutungen als auch die Anwendung von Analgetika zur Linderung von Menstruationsschmerzen reduziert [11]. Aus Daten des von 1999–2004 durchgeführten National Health and Nutrition Examination Survey fanden Cheslack-Postava und Kollegen bei 1105 Studienteilnehmerinnen keine statistisch signifikanten Hinweise auf einen Zusammenhang zwischen schwerwiegenden depressiven Syndromen und der Exposition von exogenen Geschlechtshormonen durch die Einnahme oraler Kontrazeptiva nach entsprechender Adjustierung auf relevante konfundierende Variablen [3]. Auch in ihrer kürzlich veröffentlichten Studie berichteten McKetta und Keyes bei 4765 weiblichen Jugendlichen über keinen statistisch signifikanten Zusammenhang zwischen der Einnahme oraler Kontrazeptiva und dem Auftreten von depressiven Syndromen [12].

## Aktuelle Registerdaten zu einer erhöhten Suizidalität unter hormoneller Kontrazeption

Jene Studie, die die Arzneimittelbehörden zur aktuellen Gefährdungseinschätzung mit dem vorstehend benannten Warnhinweis veranlasste, wurde in der Aprilausgabe 2018 des *American Journal of Psychiatry* von einer dänischen Arbeitsgruppe veröffentlicht [17]. Diese große epidemiologische Studie stützt sich auf landesweit in Dänemark erhobene prospektive Daten aus den Jahren 1996–2013. Die zusammengeführten Datensätze erlaubten über die Personalidentifizierungsnummern eine sichere Zuordnung aller dänischen Studienteilnehmerinnen und gestatteten den Abgleich unterschiedlicher, national erhobener Register, einschließlich zum Verordnungsverhalten von hormonalen Kontrazeptiva [17]. Bei einer durchschnittlichen Nachverfolgung über 8,3 Jahre wurden 3,9 Mio. Personenjahre bei einem Altersmittelwert der Untersuchungspopulation von 21 Jahren erfasst und in diesem Zeitraum insgesamt 6999 erste Selbstmordversuche und 71 realisierte Selbstmorde registriert. Verglichen mit Frauen, die nie hormonelle Verhütungsmittel benutzt haben, war das relative Risiko für Suizidversuche in der Gruppe der gegenwärtigen und früheren Nutzerinnen oraler Kombinationspräparate mit einer Odds-Ratio (OR) von 1,97 bei einem 95%igen Konfidenzintervall (95 %-KI) von 1,85–2,10 erhöht, ebenso wie für Suizide (OR = 3,08; 95 %-KI = 1,34–7,08). Aufgrund des Designs dieser Beobachtungsstudie konnte allerdings nicht geklärt werden, ob es sich hierbei um pharmakologisch induzierte Effekte als Folge der Exposition von Hormonpräparaten im Sinne einer medikamentös bedingten Verschlechterung der Stimmungslage handelte. Alternativ wäre es genauso denkbar, dass Frauen, die hormonelle Verhütungsmethoden praktizieren, gegenüber solchen mit nichtpharmakologischen Methoden einer Schwangerschaftsverhütung bestimmte, prädisponierende Persönlichkeitsmerkmale aufweisen, die per se mit einer erhöhten Suizidrate einhergehen.

Eine ähnliche Beziehung zwischen dem Gebrauch von Hormonpräparaten und der Selbstmordrate wurde im Register der Koreanischen Nationalen Umfrage zur Gesundheits- und Ernährungsprüfung (KNHANES) bei 27.067 Frauen im Alter von mindestens 20 Jahren beschrieben [9]. Daten aus der vom Robert-Koch-Institut durchgeführten Studie zur Gesundheit von Kindern und Jugendlichen in Deutschland (KiGGS) ergaben, dass der Gebrauch hormoneller Kontrazeptiva bei minderjährigen Mädchen mit einem erhöhten kardiovaskulären Gesundheitsrisiko einhergeht und dass dieses Risiko schädliche Verhaltensweisen wie Rauchen einschließt [5]. Da aber bislang noch keine randomisierte klinische Studie mit ausreichend großem Stichprobenumfang zur Klärung dieser wichtigen Forschungsfrage vorliegt, müssen sämtliche Aussagen über einen möglichen kausalen Zusammenhang zwischen einer Hormongabe zu Verhütungszwecken und einer gesteigerten Suizidrate notwendig spekulativ bleiben.

## Lebensqualität und psychische Auffälligkeiten bei minderjährigen Anwenderinnen hormoneller Kontrazeptiva

Sollte aber die Annahme einer Kausalität tatsächlich zutreffen, die von einer hormoninduzierten Verstärkung präexistent erhöhter Depressivität konsequenterweise zu einem Anstieg der Suizidalität führen würde, wie dieses etwa der Arzneimittelwarnbrief unterstellt, so müsste sich dieses in einer reduzierten gesundheitsbezogenen Lebensqualität und dem vermehrten Auftreten von psychischen Problemen bei den Anwenderinnen von oralen Kontrazeptiva niederschlagen. Doch fanden wir in einer Post-Analyse aus den Daten der bundesweiten, repräsentativen KiGGS-Studie bei 15- bis 17-jährigen Adoleszenten keine Hinweise für eine solche Vermutung. Weder in univariaten noch in adjustierten Modellen zeigte sich die Einnahme von oralen Kontrazeptiva verbunden mit einer geänderten gesundheitsbezogenen Lebensqualität, die über den KINDL-R-Fragebogen in Selbst- und Fremdeinschätzung psychometrisch erfasst wurde. Außerdem fanden sich in Eigen- oder Elterneinschätzung bestimmt über den SDQ („Strengths and Difficulties Questionnaire“)-Fragebogen keine Anhaltspunkte für ein vermehrtes Auftreten psychopathologischer Auffälligkeiten bei den Anwenderinnen von hormonellen Kontrazeptiva. Stattdessen fanden wir erhöhte Serum-Vitamin-D-Konzentrationen und geringgradig, aber signifikant erhöhte Blutdruckwerte bei den Nutzerinnen von Antibabypillen gegenüber ihren gleichaltrigen Nichtanwenderinnen. Da beide somatischen Parameter mit psychischem Wohlergehen assoziiert waren, kommt diesen deshalb möglicherweise eine protektive Bedeutung in Bezug auf hormoninduzierte Stimmungsschwankungen zu [1, 15].

Zusammenfassend lässt sich also konstatieren, dass die Studienlage zu psychopathologischen Auffälligkeiten einschließlich des Auftretens von depressiven Symptomen bei der Anwendung hormoneller Verhütungsmethoden mitnichten als geklärt angesehen werden kann. Somit haben wir es in dem Warnbericht gewissermaßen mit einer doppelt paradoxen Situation zu tun: Ein Zusammenhang zwischen hormoneller Kontrazeption und Depressivität wird behauptet, ohne dafür in der vorhandenen medizinischen Literatur auf sichere Belege zurückgreifen zu können, und – mehr noch – es sind die Hersteller dieser Arzneimittel, die diese voreilige Behauptung als eine gesicherte Erkenntnis ausgeben.

## Grenzen der Aufklärung über die Anwendung hormoneller Verhütungsmethoden

Welche Konsequenzen hat die Veröffentlichung des genannten Warnhinweises für das Aufklärungsverhalten von praktisch tätigen Ärztinnen und Ärzte, wenn sie ihren Patientinnen hormonelle Verhütungsmittel verschreiben wollen? Nach dem Wortsinn des § 630e Abs. 1 S. 2 BGB sind die Aufklärungspflichten bekanntlich sehr weit gefasst und schließen sämtliche behandlungsrelevanten Umstände wie Folgen und Risiken der Behandlung mit ein. Auf eine bestimmte Mindestwahrscheinlichkeit der Risikorealisierung soll es dabei gerade nicht ankommen (z. B. Bundesgerichtshof NJW 1980, 633, 634; OLG Koblenz MedR 2004, 501; OLG Köln VersR 2008, 1072: selbst bei „extrem seltenen Risiken“; BGHZ 126, 386, 389: „Risikodichte, die sich nur im Promillebereich bewegt“). Da sich diese Vorschrift aber ausdrücklich auf diagnostische und therapeutische Maßnahmen bezieht, ist zunächst zu fragen, ob es sich bei dem angetragenen Wunsch um Einleitung einer (ärztlich überwachten) medikamentösen Behandlung wirklich um eine Therapie im Sinne des § 630e Abs. 1 S. 2 BGB handelt. Zwar führt die Exposition von exogenen Geschlechtshormonen zu einem gravierenden, pharmakologisch gewünschten Eingriff in die Steuerung des Menstruationszyklus, doch sind es überwiegend nicht Erkrankte, sondern in der Mehrzahl gesunde Frauen im reproduktionsfähigen Alter, die den Arzt mit dem Wunsch nach einer medikamentösen Form der Schwangerschaftsverhütung aufsuchen. Im Licht der Patientenautonomie hat die ärztliche Aufklärungspflicht aber den Sinn, jedweden ärztlich motivierten Eingriff in die körperliche Unversehrtheit der eigenverantwortlichen Entscheidungsbefugnis der davon höchstpersönlich Betroffenen zu überantworten. Bekanntermaßen verpflichtet § 630c BGB die Parteien eines jeden medizinischen Behandlungsvertrags gleich welchen Inhalts zu gemeinsamem, vorteilhaftem Mitwirken, worunter im weiteren Sinn nicht nur die eigentliche Domäne der Therapie, sondern auch präventive Maßnahmen fallen, im hiesigen Beispiel also die Verhinderung einer ungewollten Schwangerschaft. Obgleich der Therapiebegriff in Bezug auf die Anwendung von hormonellen Kontrazeptiva sicherlich zu kurz greift, beinhaltet § 630e BGB eine Aufklärungsverpflichtung des Behandelnden auch bei präventiven und nicht nur bei ausschließlich kurativen Behandlungsmaßnahmen.

Damit ist im Grundsatz eine überbordende Aufklärung von Rechts wegen vorgegeben, die dem Ideal einer „vollständigen“ Informiertheit des Patienten über sämtliche „objektiv“ (d. h. medizinisch) relevanten Fakten zwecks bestmöglicher Befähigung zur selbstbestimmten Entscheidung folgt. Nach aktueller Rechtsprechung des BGH hat der aufklärende Arzt im Fall der medikamentösen Behandlung die Fachinformation nach § 11a AMG als eine wichtige Informationsquelle zu nutzen und darf davon ausgehen, dass diese nach objektiven Maßstäben unter Einschluss aller veröffentlichten Ergebnisse und auch unveröffentlichten Meldungen über das Pharmakovigilanzsystem erstellt wurde (OLG Köln MedR 2017, 250; [20]). Schließlich obliegt es den Arzneimittelbehörden, über das Pharmakovigilanzsystem erkannte, potenziell gefährliche Komplikationen durch ein entsprechendes Repertoire an Gegenmaßnahmen abzuwenden. Ihre Entscheidungen dienen ausschließlich dem Schutz der Konsumenten und rechtfertigen ein marktregulierendes Eingreifen in Gefährdungssituationen auch dann, wenn begründete wissenschaftliche Zweifel an einer Maßnahme fortbestehen oder die Evidenzlage noch nicht abschließend geklärt werden kann. Zuletzt hat die Rechtsprechung in einem Judikat des OLG Köln die haftungsrechtliche Bedeutung der Fachinformationen nochmals herausgestellt, obgleich, wie Gödicke in seiner Urteilsbesprechung zu Recht betont, seitens der Ärzteschaft wohl eine gewisse Reserviertheit hinsichtlich deren Bindungskraft bestehen dürfte, da ihnen ein Defensivcharakter für den pharmazeutischen Hersteller unterstellt wird [7]. Dass in einer Fachinformation auch nichtpublizierte Meldungen über Arzneimittelnebenwirkungen angegeben sind, deren Plausibilität für den Behandler damit nicht oder nur sehr schwer einsehbar ist, erhöht nicht gerade die Bereitschaft auf Behandlerseite, sich ganz und vollumfänglich auf die Fachinformationen zu Aufklärungszwecken zu berufen, zumal die pharmazeutischen Unternehmen die Fachinformationen auch nur auf ärztliche Anforderung zukommen lassen. Jedenfalls wird man aber zwingend eine gewisse empirische „Mindestevidenz“ für dahingehende Risikoannahmen verlangen müssen, damit nicht bloß spekulative Mutmaßungen Eingang in die Patientenaufklärungen finden.

Dies gilt umso mehr, als auch der Behandlungsstandard als Bezugspunkt der Patienteninformation (regelmäßig) eine – sogar hohe – wissenschaftliche und klinische Evidenz verlangt, d. h. eine Gesamtschau der medizinischen Literatur und Erfahrungen impliziert und nicht von singulären Studienergebnissen bestimmt wird. Maßgeblich ist vielmehr dasjenige, was in Leitlinien medizinischer Fachgesellschaften Eingang gefunden hat, auch wenn die darin enthaltenen Handlungsanweisungen selbstredend im jeweiligen Krankheitsfall nicht blind exekutiert werden dürfen. Abweichungen von der empfohlenen Leitliniendosis sind dann nicht haftungsrelevant, wenn sie in der medizinischen Fachliteratur an anderer Stelle als durchaus therapieförderlich gekennzeichnet werden [23]. Auf die Aufklärungserfordernisse bezogen kann deshalb davon ausgegangen werden, dass – wie hier am Beispiel der oralen Kontrazeptiva gezeigt – einzelne Studienergebnisse ohne Einbettung in eine konsistente Erkenntnislage im Ganzen nicht geeignet sind, über eine notwendige Sensibilisierung der Behandlerseite hinaus ungefiltert auf die Information des Patienten durchschlagen können, und dies umso mehr, wenn der Warnhinweis unter Umständen sogar sachlich fehlerhaft oder zumindest in der Darstellung einseitig ist. Die Warnhinweise der Arzneimittelbehörden unterliegen weniger umfangreichen und strengen Plausibilitätskontrollen, als sie üblicherweise bei der Erstellung von Leitlinien oder wissenschaftlichen Publikationen eingefordert werden. Zudem findet ein bei der Veröffentlichung klinischer Studien oder Beobachtungen als wissenschaftliches Qualitätskriterium üblicher Peer-Review-Prozess bei der Veröffentlichung von Warnhinweisen nicht statt.

Darüber hinaus besteht bei Verordnung eines Arzneimittels keineswegs unbegrenzt eine Aufklärungspflicht des jeweils behandelnden Arztes; denn nicht ohne Grund ist bereits der pharmazeutische Hersteller nicht bloß zur detaillierten Fachinformation an Ärzte etc. (§ 11a AMG), sondern ebenso zur sorgfältigen Erstellung der unmittelbar patientenbezogenen „Gebrauchsinformationen“ (sog. Beipackzettel) verpflichtet (§ 11 AMG). Schon hierdurch soll der jeweilige Patient zur sachgerechten Anwendung des Arzneimittels befähigt werden, u. a. auch durch Hinweise auf evtl. Gegenanzeigen und Nebenwirkungen bei bestimmungsgemäßem Gebrauch. Normativ fällt es demzufolge in die Sphäre seiner Eigenverantwortung, diese Informationen vor Einnahme des Medikaments sorgfältig zur Kenntnis zu nehmen, mag dies in praxi auch weithin vernachlässigt werden. Spiegelbildlich entlastet diese Verantwortungszuschreibung an die Patienten den jeweiligen behandelnden Arzt, weil dieser grundsätzlich davon ausgehen darf, dass seinem Gegenüber bereits eine seriöse Informationsquelle ungehindert zugänglich ist. Nicht ohne Grund zielt die Pflicht des Herstellers auf „allgemein verständliche“ Angaben (§ 11 Abs. 1 S. 1 AMG), die also auch für medizinische Laien bestmöglich verstehbar sind.

Gleichwohl ist nach vorherrschender Rechtsauffassung der im Einzelfall behandelnde Arzt nicht vollständig aus seiner arzneimittelbezogenen Aufklärungspflicht befreit. Der BGH hat vielmehr herausgestellt, dass bei schwerwiegenden Nebenwirkungen eines Medikaments wie bei der Verordnung eines hormonbasierten Antikonzeptionsmittels zur Behandlung einer Dysmenorrhö (in dem zugrunde liegenden Fall) neben dem Hinweis in der Gebrauchsinformation des Pharmaherstellers auch eine umfassende Aufklärung über seltene Nebenwirkungen durch den das Medikament verordnenden Arzt erforderlich ist (NJW 2005, 1716). Bei solchermaßen erheblichen Folgen und Risiken könne der Arzt somit nicht mehr einfach auf die Selbstverantwortung des Patienten vertrauen, sondern müsse diese Dimension einer Medikamenteneinnahme nochmals zum Gegenstand auch seines persönlichen (nach § 630e Abs. 2 Nr. 1 BGB grundsätzlich mündlichen) Aufklärungsgesprächs machen. Aufgeklärt werden muss aber auch dann nicht etwa über den gesamten Inhalt des Beipackzettels, sondern allein über dasjenige, worauf es im individuellen Krankheitsfall ankommt [4]. Die Beweislast hierfür trifft den verordnenden Arzt, der sich nicht durch Verweis auf die Gebrauchsinformation des Pharmaherstellers exkulpieren kann. Einen Schadensersatzanspruch begründet die Verletzung der ärztlichen Aufklärungspflicht allerdings nur dann, wenn hernach eine bekannte Nebenwirkung auftritt, im erwähnten BGH-Fall ein apoplektischer Insult bei einer Raucherin, die – wissenschaftlich bewiesen – als kausale Folge der Medikamenteneinnahme gelten kann. Sollte im Einzelfall ein schweres Folgerisiko bestehen, so muss der Arzt auch dann darüber aufklären, wenn nach allgemeinem Beurteilungsmaßstab diese Komplikation nur sehr selten auftritt, für den individuellen Patienten aber mit einer starken Gesundheitsbeeinträchtigung einhergeht.

Insgesamt ergibt sich daher: Solange innerhalb der einschlägigen Fachliteratur weithin kontrovers diskutiert [13], hat eine unterbliebene Aufklärung für eine möglicherweise erhöhte Suizidalität durch hormonelle Kontrazeptiva keine juristischen Konsequenzen. Denn wenn wegen fehlenden Nachweises einer solide begründeten Expertenmeinung oder wegen der aktuell noch unzureichenden Evidenzlage von einem erkannten Risiko gegenwärtig nicht ausgegangen werden kann, fehlt es an der hinreichenden Validität, um zum notwendigen Gegenstand des Aufklärungsgesprächs zu werden. Geboten ist dann nicht ein Spekulieren, sondern das Unterlassen von Spekulationen.

## Fazit für die Praxis

- § 630e BGB beinhaltet von Rechts wegen eine im Grundsatz überbordende ärztliche Aufklärungspflicht, die dem Ideal einer vollständigen Informiertheit des Patienten über sämtliche medizinisch relevanten Fakten zwecks bestmöglicher Befähigung zur selbstbestimmten Entscheidung zugrunde liegt.
- Eine umfassende Aufklärung bedarf grundsätzlich auch der Erwähnung seltener Arzneimittelwirkungen durch den verordnenden Arzt, sollten diese im Einzelfall schwerwiegende Folgen haben.
- § 630e BGB sieht eine Aufklärungsverpflichtung des Behandelnden auch bei präventiven und nicht nur bei ausschließlich kurativen Behandlungsmaßnahmen vor.
- Der über eine medikamentöse Behandlung aufklärende Arzt hat die Fachinformation nach § 11a AMG als eine wichtige Informationsquelle zu nutzen und darf davon ausgehen, dass diese nach objektiven Kriterien erstellt wurde.
- Aktuelle Warnhinweise der Arzneimittelbehörden in Form von Rote-Hand-Briefen haben jedoch nicht zwingend juristische Konsequenzen für die Inhalte des ärztlichen Aufklärungsgesprächs.
